# Combined use of multiparametric high-content-screening and in vitro circadian reporter assays in neurotoxicity evaluation

**DOI:** 10.1007/s00204-024-03686-6

**Published:** 2024-03-14

**Authors:** Youngil Park, Hwan-Goo Kang, Seok-Jin Kang, Hyun-Ok Ku, Helmut Zarbl, Ming-Zhu Fang, Jae-Hak Park

**Affiliations:** 1 Laboratory Animal Medicine, College of Veterinary Medicine, Seoul National University, Seoul, 08826 Korea; 2 Veterinary Drugs and Biologics Division, Animal and Plant Quarantine Agency, Gimcheon-Si, 39660 Korea; 3 Department of Environmental and Occupational Health, School of Public Health, NIEHS Center for Environmental Exposure and Disease, Environmental and Occupational Health Sciences Institute, Rutgers, The State University of New Jersey, Piscataway, NJ08854 USA; 4 Present Address: Department of Animal Health and Welfare, Semyung University, 65, Semyung Ro, Jecheon, Chungcheongbuk‑do Korea

**Keywords:** Circadian rhythm, High-content screening, In vitro bioluminescence assay, Neurotoxicity, PER2

## Abstract

Accumulating evidence indicates that chronic circadian rhythm disruption is associated with the development of neurodegenerative diseases induced by exposure to neurotoxic chemicals. Herein, we examined the relationship between cellular circadian rhythm disruption and cytotoxicity in neural cells. Moreover, we evaluated the potential application of an in vitro cellular circadian rhythm assay in determining circadian rhythm disruption as a sensitive and early marker of neurotoxicant-induced adverse effects. To explore these objectives, we established an in vitro cellular circadian rhythm assay using human glioblastoma (U87 MG) cells stably transfected with a circadian reporter vector (*PER2*-dLuc) and determined the lowest-observed-adverse-effect levels (LOAELs) of several common neurotoxicants. Additionally, we determined the LOAEL of each compound on multiple cytotoxicity endpoints (nuclear size [NC], mitochondrial membrane potential [MMP], calcium ions, or lipid peroxidation) using a multiparametric high-content screening (HCS) assay using transfected U87 MG cells treated with the same neurotoxicants for 24 and 72 h. Based on our findings, the LOAEL for cellular circadian rhythm disruption for most chemicals was slightly higher than that for most cytotoxicity indicators detected using HCS, and the LOAEL for MMP in the first 24 h was the closest to that for cellular circadian rhythm disruption. Dietary antioxidants (methylselenocysteine and *N*-acetyl-l-cysteine) prevented or restored neurotoxicant-induced cellular circadian rhythm disruption. Our results suggest that cellular circadian rhythm disruption is as sensitive as cytotoxicity indicators and occurs early as much as cytotoxic events during disease development. Moreover, the in vitro cellular circadian rhythm assay warrants further evaluation as an early screening tool for neurotoxicants.

## Introduction

Neurodegenerative diseases have been associated with the disruption of daily rhythms (Carter et al. 2021; Logan and McClung 2019; Nassan and Videnovic 2022). According to human and rodent studies, circadian rhythm disruption can be attributed to shift work, light exposure at night, pinealectomy, or genetic defects (e.g., lack of circadian genes) (Antoch et al. 2008; Fu and Lee 2003; Kondratov et al. 2006; Lee et al. 2010; National Toxicology 2021; Salgado-Delgado et al. 2011). Numerous clinical studies have revealed a direct correlation between abnormal clock function and the severity of neurodegeneration and sleep disturbances (Hastings and Goedert 2013; Leng et al. 2019; Shen et al. 2023). Furthermore, animal models of Alzheimer’s disease (Sterniczuk et al. 2010), Parkinson’s disease (Kudo et al. 2011), and Huntington’s disease (Oakeshott et al. 2011) have confirmed the adverse effects of circadian clock disruption on neurodegeneration.

Investigations exploring neurological diseases using laboratory animal models are costly, time-consuming, and unsuitable for screening large numbers of chemicals. Moreover, in vivo studies lack adequate sensitivity to predict human neurotoxicity and often fail to generate sufficient information for regulatory decision-making (Bal-Price et al. 2008). Therefore, it is imperative to establish and validate cell-based in vitro assays to monitor changes in cell-autonomous rhythms using initial screening methods for neurotoxic chemicals, given that neurotoxicity prediction is an important feature of toxicological profiling of chemical compounds and environmental toxicants. Furthermore, chemicals responsible for various diseases have been shown to alter cellular circadian rhythm (Audira et al. 2019; Boughattas et al. 1989; Chen et al. 2012; Fifel and Videnovic 2020; Hwang et al. 2014; Wilsbacher et al. 2002; Wong and Cortopassi 2002); Neurotoxicity induced by external factors, such as chemicals, can result in disease, with major toxicity mechanisms including excess nitrogen oxide production, reactive oxygen species (ROS)-induced oxidative stress, mitochondrial dysfunction (Amo et al. 2011; Zerin et al. 2015), calcium channel dysfunctions (Baev et al. 2022), and DNA damage (Zhang 2018); these factors have been found to modulate cellular circadian rhythm (Cavieres-Lepe and Ewer 2021; Fagiani et al. 2022; Fanjul-Moles and López-Riquelme 2016). Therefore, the impact of cellular circadian rhythm disruption on toxicological effects is crucial for assessing the neurotoxicity of chemical compounds and environmental toxicants.

The characteristics of the cellular circadian rhythm have been investigated in the suprachiasmatic nucleus of various laboratory animal species (Brown et al. 2019; Rivkees 2007). Importantly, the existence of the circadian rhythm has been confirmed in peripheral tissues and cells (Balsalobre et al. 1998), such as fibroblasts (Izumo et al. 2003), and changes in cellular circadian rhythm following chemical exposure have been documented and quantitatively analyzed (Hirota and Kay 2009). Cell experiments have shown that circadian rhythm disruption is associated with shortened or prolonged circadian periods, increased or decreased amplitude, and/or advanced or delayed rhythmic expression of circadian rhythm-related genes (Chen et al. 2012; Izumo et al. 2006). Subsequently, in vitro tests were developed to analyze the circadian rhythm of the liver (Guenthner et al. 2014; Saini et al. 2013; Yang et al. 2010), brain (Slat et al. 2017), and mammary gland (Fang et al. 2017) cells.

Alterations in the circadian rhythm in response to environmental chemicals have been studied in both in vivo and in vitro models (Grill and Maganti 2011; Richardson et al. 2019). For example, an in vitro investigation using liver cells to examine changes in the cellular metabolic rhythms of environmental substances has been reported, along with a review paper exploring cellular rhythm in zebrafish in relation to environmental pollutants (Ndikung et al. 2020; Zheng et al. 2021). However, properties that induce cellular circadian rhythm disturbance remain poorly established for several of these substances. The molecular mechanisms underlying changes in the cellular circadian rhythm have been identified through animal experiments and in vitro tests (Fang et al. 2015; Partch et al. 2014).

In the present study, we aimed to explore the relationship between disruption of the cellular circadian rhythm and cytotoxicity in neural cells. Accordingly, we combined in vitro circadian rhythm reporter assays and multiparametric high-content screening (HCS), which provides a mechanistic basis for effortless neurotoxicity screening based on different cellular functions without intending to study the detailed molecular biological mechanisms. We selected neurotoxicants known to cause oxidative stress (e.g., chlorpyrifos [CPF] and 1-methyl-4-phenylpyridinium [MPP +]) (Li et al. 2021; Singh et al. 2018), mitochondrial damage/dysfunction (e.g., CPF) (Yamada et al. 2017), and calcium channel inhibition (e.g., 4,4′-dichlorodiphenyltrichloroethane [DDT]) (Costa et al. 2008), as well as antibiotics capable of inducing neurotoxic effects (e.g., ciprofloxacin [CPX]; polymyxin B [PMX]) (Grill and Maganti 2011; Xiao et al. 2019), and heavy metals (e.g., methylmercury (II) chloride [MHg]) (Davidson et al. 2004; Yuan and Atchison 2016) as environmental contaminants, and tested these compounds in an in vitro assay using U87 MG cells. Comparing the chemical concentrations that induce cellular circadian rhythm changes with those that alter different mechanistic endpoints of cytotoxicity can provide valuable insights into the association between cellular rhythm disruption and general neurotoxicity.

## Materials and methods

### Materials

MHg was purchased from Sigma-Aldrich (442534). Additionally, we procured the following pesticides: CPF (45395; Sigma-Aldrich); MPP + iodide (N048; Sigma-Aldrich); DDT (N10876; Sigma-Aldrich). We also purchased the following antibiotics: PMX sulfate salt (Sigma P4932; Sigma-Aldrich) and CPX (17850; Sigma-Aldrich). Depending on the solvent employed for chemical dissolution, a vehicle control group was established: vehicle control: 0.1% dimethyl sulfoxide (DMSO [D12345; Invitrogen])-treated media or naïve media. For example, MPP + , PMX, and CPX were dissolved in distilled water or 0.1 N HCl and stored in aliquots at concentrations of 30 mM or 20 mM; MHg, DDT, and CPF were dissolved in DMSO and stored in aliquots with concentrations of 5 mM or 10 mM, and then diluted in a medium for experimental use.

### Establishment of an in vitro cellular bioluminescence assay using U87 MG neural cells

U-87 MG (ATCC® HTB-14™) is a well-recognized human glioblastoma cell line widely used in the field of neuroscience. U87 MG cells have a regular and strong cellular circadian rhythm after transfection with a circadian gene (i.e., *BMAL1*) reporter vector (Jung et al. 2013; Slat et al. 2017). As described in our previous report (Fang et al. 2015, 2017), U87 MG cells were transfected with a circadian reporter vector pGL [*PER*2P (Luc2P/Neo)] expressing destabilized firefly luciferase driven by the human *PER*2 promoter using FuGene HD™ transfection reagent (LightSwitch Genomics), and stably transfected cells were selected and cultured with 1000 μg/mL G418 (Invitrogen). The stably transfected cells were used to examine the cellular circadian rhythm using an in vitro cellular bioluminescence assay. U87 MG/*PER*2P-dLuc cells were cultured in Eagle’s minimum essential medium (EMEM) (11430030; Gibco) supplemented with 5% fetal bovine serum (FBS) (26140079; Gibco) and 1% penicillin/streptomycin (15140122; Gibco) at 37 °C in an atmosphere of 95% humidity and 5% CO_2_.

### Cell viability assay

U87 MG/*PER*2-dLuc cells were subjected to the 3-(4, 5-dimethylthiazol-2-yl)-2, 5-diphenyl tetrazolium bromide (MTT) (M2128; Sigma-Aldrich) assay. To determine whether *PER*2P influences cell toxicity and establish working concentrations of the chemicals used in the current study (concentrations: MHg [1–9 µM]; CPF [20–180 µM]; MPP + [1 mM–1000 mM]; DDT [100–1000 µM]; PMX [0.1–1.0 mM]; CPX [0.76–100 µM]), we performed MTT cell viability assays and calculated the IC_50_/IC_15_ inhibitory concentrations of transfected cells. Briefly, U87 MG/*PER*2-dLuc cells were seeded in 96-well plates at a density of 10^5^ cells/mL, incubated for 48 h, and treated with relevant chemicals at various concentrations for 72 h. Subsequently, the media with chemicals were decanted and 20μl of MTT reagent solution (5 mg/ml) with 80μl of the media was added to all wells the plates and were incubated for 2 h until the formation of formazan crystals. The reagent solution was decanted, and 100μl of DMSO (D8418; Sigma-Aldrich) was added. Absorbance was measured at 570 nm using a spectrophotometer (Molecular Devices). The values of triplicate same-treatment wells were averaged. For each compound, concentrations resulting in 15% (IC_15_) and 50% (IC_50_) growth inhibition were determined using GraphPad Prism 9.0 (GraphPad Software, Inc., La Jolla, CA, USA).

### Assessment of the effects of neurotoxicants on cellular circadian rhythm

Briefly, cultured U87 MG/*PER*2 -dLuc cells were seeded in 35-mm dishes at 3 × 10^5^ cells/dish and incubated until 70–80% confluency was reached. The cells were FBS-starved for three days at 70% confluency and then synchronized with 50% horse serum (16050122; Gibco) (Balsalobre et al. 1998). After decanting and washing, recording medium (EMEM with 20% of growing medium containing 5% FBS, 6.5 mM sodium bicarbonate, 10 mM HEPES buffer [pH 7.2], 0.1 mM luciferin [E1602; Promega], and 50 U/mL penicillin and 50 µg/mL streptomycin) was added. Subsequently, the cells were treated with each compound. The 35-mm dishes were sealed with a sterile glass cover slide using silicon grease and subjected to continuous monitoring with a LumiCycle 32™ (Actimetrics) for 5 days. The analytical method was optimized according to the instruction manual of the LumiCycle Analysis Software (Actimetrics). Using this software, data were detrended (running average), and best fits to a sine wave were estimated using the Levenberg–Marquardt algorithm for period, phase, amplitude, and damping rate measurements, as previously reported (Chen et al. 2012; Fang et al. 2015, 2017).

### Assessment of the effects of neurotoxicants on cellular circadian rhythm in the presence of antioxidants

To determine the effects of antioxidants, U87 MG/*PER*2-dLuc cells were treated as previously described with neurotoxicants (MHg, CPF, MPP + , DDT, PMX, or CPX) at respective lowest-observed-adverse-effect levels (LOAELs), along with one of the following antioxidants: 12.5 or 25 µM methylselenocysteine (MSC) (M6680; Sigma-Alrich) or 10 or 20 µM *N*-acetyl-l-cysteine (NAC) (A7250; Sigma-Alrich) added in the recording medium, as previously described. The cellular circadian rhythm was recorded as described above.

### HCS assay

A multiparametric analysis of compound toxicity at the level of individual cells was conducted using the HCS method established by Tolosa et al. (2012) to assess the liver toxicity of chemicals and drugs. Briefly, U87 MG/*PER*2-dLuc cells were seeded in 96-well plates specific for HCS at a density of 10^4^ cells/well. The cells were incubated with the chemicals at various concentrations (concentrations: MHg [0.03–8 µM]; CPF [0.7–180 µM]; MPP + [0.6 µM–160 µM]; DDT [0.5–120 µM]; PMX [4–1000 µM]; CPX [0.9–240 µM]) for 24 or 72 h. Following these treatments, cells were treated with a mixture of Hoechst 33342 (Hoechst) (B2261; Sigma-Alrich) at 1.5 µg/mL to assess the nuclear size (NC), 75 ng/mL tetramethyl rhodamine methyl ester (TMRM) (T-668; Molecular Probes) as a mitochondrial membrane potential (MMP) sensor, 0.24 µM fluo-4 acetoxymethyl ester (Fluo-4 AM) (F14217; Molecular Probes) to detect intracellular calcium ions (Ca^2+^), and 1.5 µg/mL of (E,E)-3,5-bis-(4-phenyl-1,3-butadienyl)-4,4-difluoro-4-bora-3a,4a-diaza-s-indacene (BODIPY 665/676) (b-3932; Molecular Probes) to evaluate lipid peroxidation. After incubation for 1 h, the cells were imaged using an IN Cell Analyzer 6000 (GE Healthcare Life Sciences) and analyzed using IN Cell Miner HCM. The data were analyzed with IN Cell Analyzer 6000 Analysis Software. Nuclear morphological alterations were assessed based on the Hoechst staining. The nucleus was defined as the main object detected using an edge detection algorithm. To separate individual cells, segmentation was applied. All measurements were restricted to live cells. Lipid peroxidation was detected based on BODIPY fluorescence intensity in the cytoplasm. The cellular MMP was defined as the TMRM fluorescence intensity in punctate cytosolic regions surrounding the nucleus. Fluo-4 intensity was used to measure changes in the cytosolic-free Ca^2+^ concentration. An intensity algorithm with a fixed threshold was used to measure TMRM, Fluo-4, and BODIPY fluorescence. Each measurement was performed in individual cells. The values of triplicate same-treatment wells were averaged and normalized to the average value of vehicle control (0.1% DMSO-treated or naïve cells).

### Statistical analysis

The values of triplicate same-treatment wells were averaged and normalized to the average value of the negative control group, and differences in parameters between concentrations were analyzed using one-way ANOVA followed by Duncan’s tests. A *p*-value < 0.05 was considered significant.

## Results

### Toxicity of chemicals in U87 MG/*PER*2-dLuc cells

**Fig. 1:**
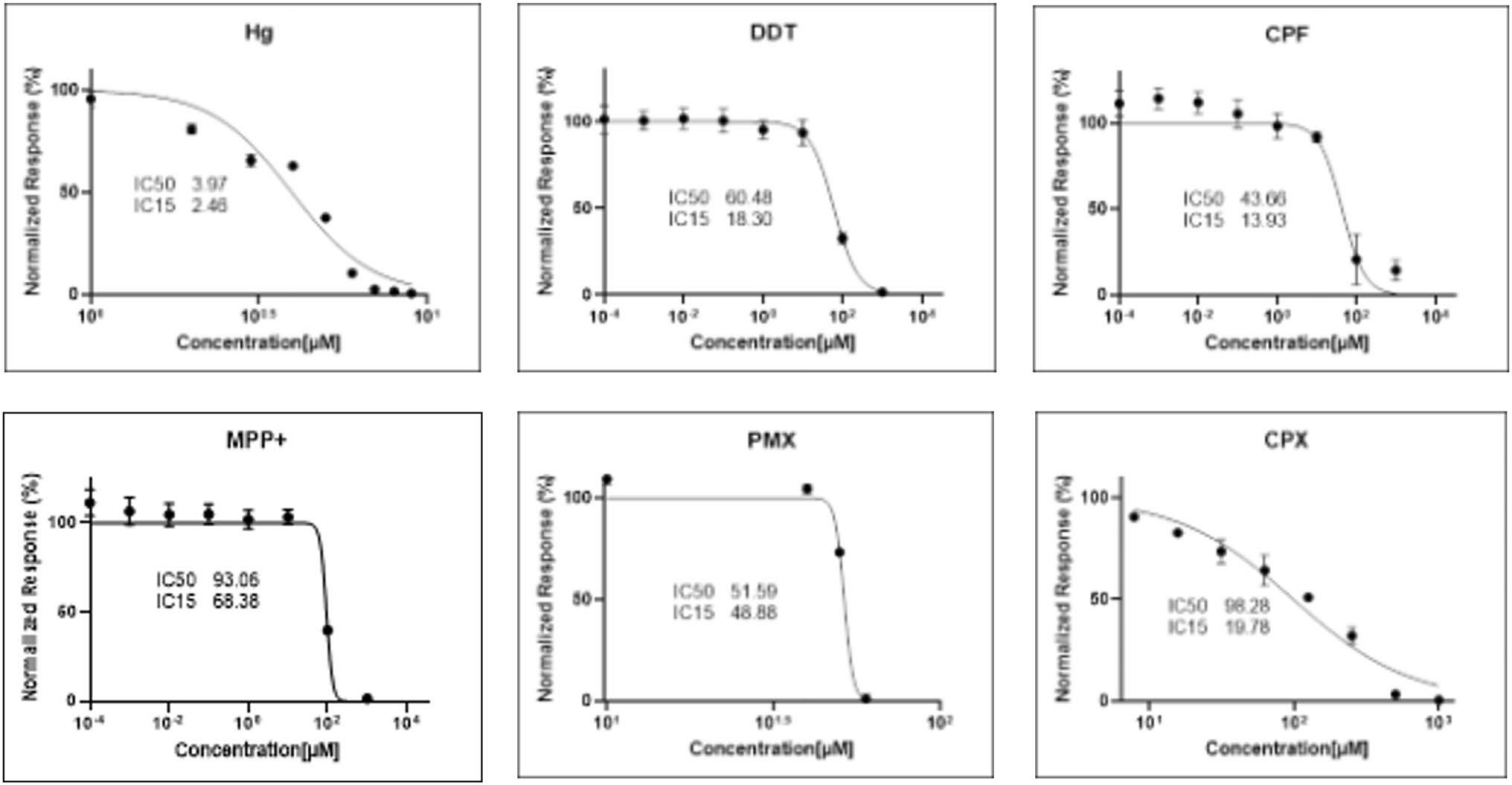
Cell viability of U87 MG/*PER*2-dLuc cells post-treatment. U87 MG/*PER*2-dLuc cells were treated with each compound at the indicated doses, and cell viability was examined using the MTT assay. The IC_50_–IC_15_ concentration range of each compound was estimated using GraphPad Prism 9.0 (GraphPad). CPF, chlorpyrifos; CPX, ciprofloxacin; DDT, 4,4′-dichlorodiphenyltrichloroethane; MHg, methylmercury; MPP + ,1 -methyl-4-phenylpyridinium; PMX, polymyxin B

To determine the concentration range of each chemical to be employed in the in vitro cellular bioluminescence assay and HCS, we measured the viability of U87 MG/*PER*2-dLuc cells treated with individual compounds using the MTT assay. Cell viability as a function of concentration is presented in Fig. 1. Among the examined compounds, MHg exhibited the highest toxicity toward U87 MG/*PER*2-dLuc cells, with an IC_50_ of 3.97 µM, whereas CPX exerted the lowest toxicity (IC_50_ = 98.3 µM). The order of toxicity was as follows: MHg > DDT > MPP +  > CPF > PMX > CPX. For each examined compound, concentrations covering the IC_50_–IC_15_ range were used in the in vitro cellular bioluminescence assay.

### Cellular circadian rhythm of U87 MG cells transfected with a circadian reporter vector and toxicity of chemicals on transfected U87 MG/*PER*2-dLuc cells

**Fig. 2:**
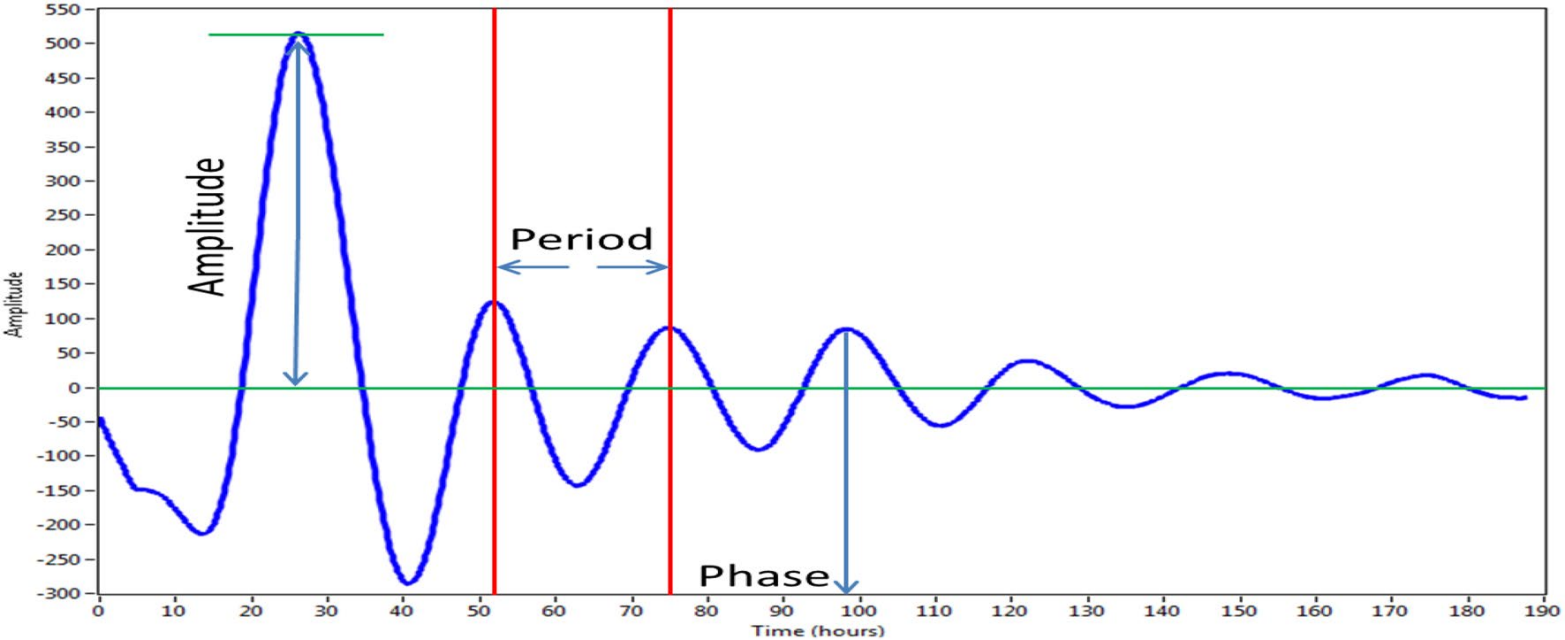
Representative circadian rhythm of U87 MG/*PER*2-dLuc reporter cells in the in vitro cellular bioluminescence assay. Human glioblastoma cells (U-87 MG) were stably transfected with a circadian reporter vector. U87 MG*/PER*2-dLuc cells show a regular and strong cellular circadian rhythm

The cellular circadian rhythm was evaluated based on circadian parameters, including amplitude, period, and phase, as described previously (Fang et al. 2015, 2017). U87 MG cells stably transfected with the circadian reporter vector (U87 MG/*PER*2-dLuc) exhibited a regular and strong cellular circadian rhythm for at least 7 days (Fig. 2), comparable with that observed in human mammary epithelial cells (Fang et al. 2015, 2017). This finding indicated that our circadian luciferase reporter vector and in vitro cellular bioluminescence assay could be applied to U87 MG human neural cells.

### Disruption of the circadian rhythm in the in vitro cellular bioluminescence assay using U87 MG/*PER*2-dLuc neural cells

**Fig. 3:**
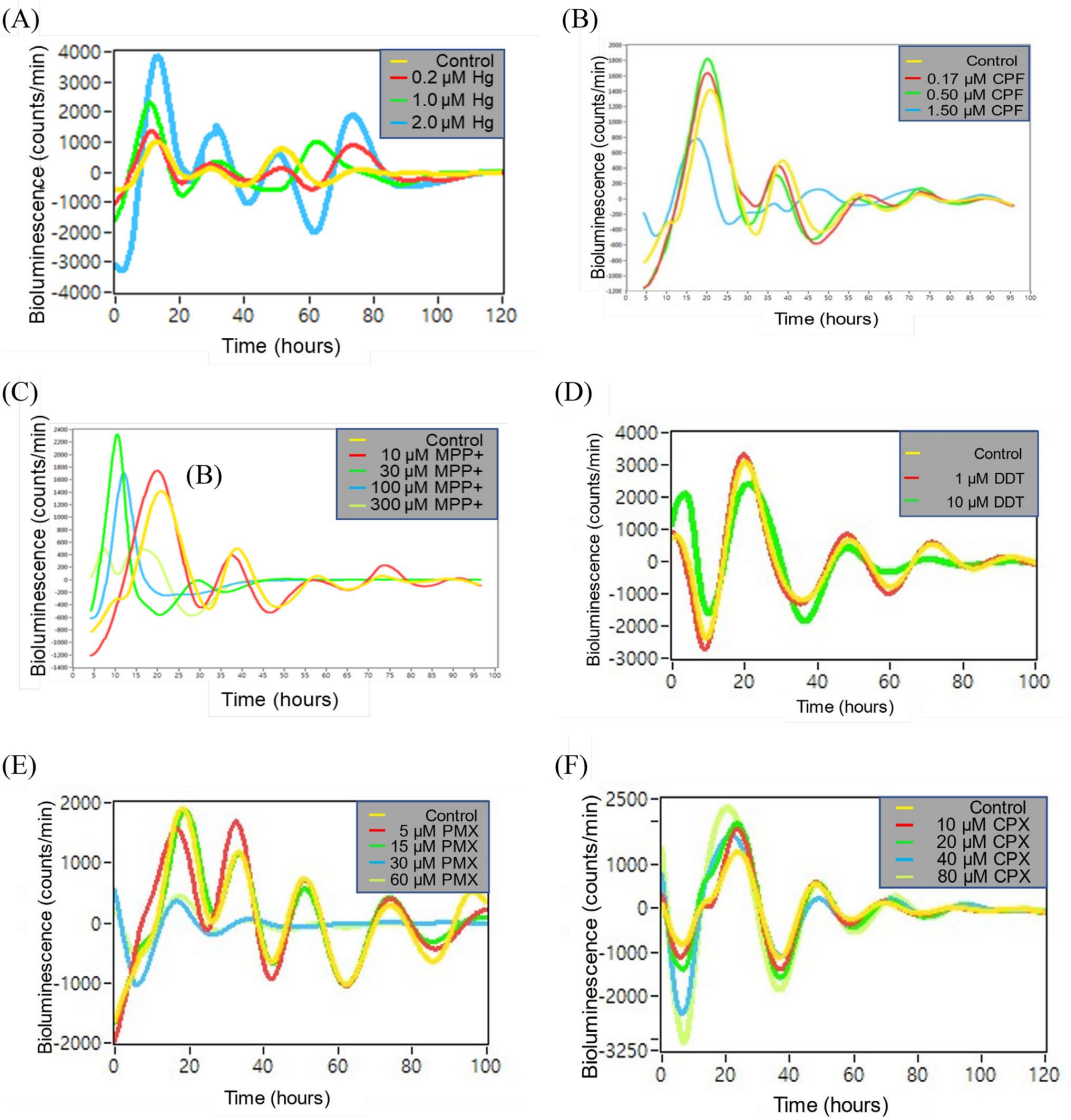
In vitro cellular circadian rhythms of U87 MG/*PER2*-dLuc cells disrupted by neurotoxicants. Bioluminescence assays were performed for each treatment using U87 MG/*PER*2-dLuc reporter cells after synchronization with 50% horse serum. X-axis, time (hours) post-chemical treatment; Y-axis, bioluminescence (count/min). (A) MHg, (B) CPF, (C) MPP + , (D) DDT, (E) PMX, and (F) CPX. CPF, chlorpyrifos; CPX, ciprofloxacin; DDT, 4,4′-dichlorodiphenyltrichloroethane; MHg, methylmercury; MPP + ,1 -methyl-4-phenylpyridinium; PMX, polymyxin B

According to bioluminescence assay results, 2.0 µM MHg (Fig. 3A) and 1.5 µM CPF (Fig. 3B) induced circadian rhythm disruption of U87 MG/*PER*2-dLuc neural cells. Additionally, MPP + disrupted the cellular circadian rhythm at concentrations ≥ 30 µM, with an advanced phase and decreased amplitude, although no disruptive effect was observed at a lower concentration (10 µM) (Fig. 3C). DDT appeared to be well tolerated except at the highest concentration (10 µM), at which the robust rhythm was gradually abolished (Fig. 3D). PMX disrupted the cellular circadian rhythm at concentrations ≥ 30 µM, with an advanced phase and decreased amplitude, but showed no disruptive effect at a lower concentration (15 µM) (Fig. 3E). CPX increased the circadian rhythm at concentrations ≥ 40 µM. After treatment with ≥ 40 µM CPX, reporter activities were enhanced during the first circadian cycle and persisted even in subsequent cycles (Fig. 3F). Table 1 summarizes the effects of all examined compounds on cellular circadian rhythm.

**Table 1:** Comparison of the LOAELs of the cellular circadian rhythm with those of each cytotoxic endpoint detected in U87 MG/*PER*2-dLuc cells after 24 or 72 h of treatment (unit: µM)

Compound (IC_15_, IC_50_)	Circadian disruption	24-h treatment				72-h treatment			
		NC	MMP	CA	LP	NC	MMP	CA	LP
MHg (2.5, 3.9)	**1.0**	4**	0.5*	NA	8**	4**	NA	0.031**	0.031**
CPF (13.9, 60)	**1.5**	NA	1.41**	1.41**	NA	NA	NA	0.7**	NA
MPP + (68.4, 93)	**30**	ND	40*	40	NA	80	NA	**0.63**	160
DDT (18.3, 60)	**10**	120**	15	30**	NA	NA	NA	0.47**	NA
PMX (49, 51)	**30**	NA	3.91*	ND	NA	NA	NA	3.91**	NA
CPX (19.8, 98)	**40**	NA	0.94**	ND	NA	NA	ND	0.94**	0.94**

The LOAEL of the cellular circadian rhythm was determined in U87 MG/*PER*2-dLuc cells using an in vitro cellular bioluminescence assay. The IC_15_ or IC_50_ values of cytotoxicity endpoints were determined in U87MG/*PER*2-dLuc cells after treatment with each chemical for 72 h using the MTT assay. Chemical concentrations that showed significant values with HCS were analyzed using ANOVA, followed by Duncan’s tests. *p < 0.05 or **p < 0.01 represent significant differences compared with the vehicle control (0 µM) at each time point, 24 or 72 h. **bold**, LOAEL associated with circadian rhythm disturbance or cytotoxic changes in HCS; CPF, chlorpyrifos; *CPX* ciprofloxacin, *DDT* 4,4’-dichlorodiphenyltrichloroethane, *HCS* high-content-screening, *LOAEL* lowest-observed-adverse-effect level, *MHg* methylmercury, *MMP* mitochondrial membrane potential, *MPP + *1 -methyl-4-phenylpyridinium, *PMX* polymyxin B, *NC* nuclear size, *LP* lipid peroxidation, *CA* calcium ion, *NA* not applicable, *ND* not determined

### Antioxidants restored the chemical-induced circadian rhythm disruption

The dietary antioxidant agents MSC and NAC are known to reduce oxidative stress and prevent the loss of or restore the normal pattern of cellular circadian rhythm (Fang et al. 2015). To determine whether disrupted cellular circadian rhythms are associated with oxidative stress, we simultaneously administered NAC (10 or 20 μM) or MSC (12.5 or 25 μM) at the minimum disrupted concentration for each chemical, as previously reported (Fang et al. 2017).

**Fig. 4:**
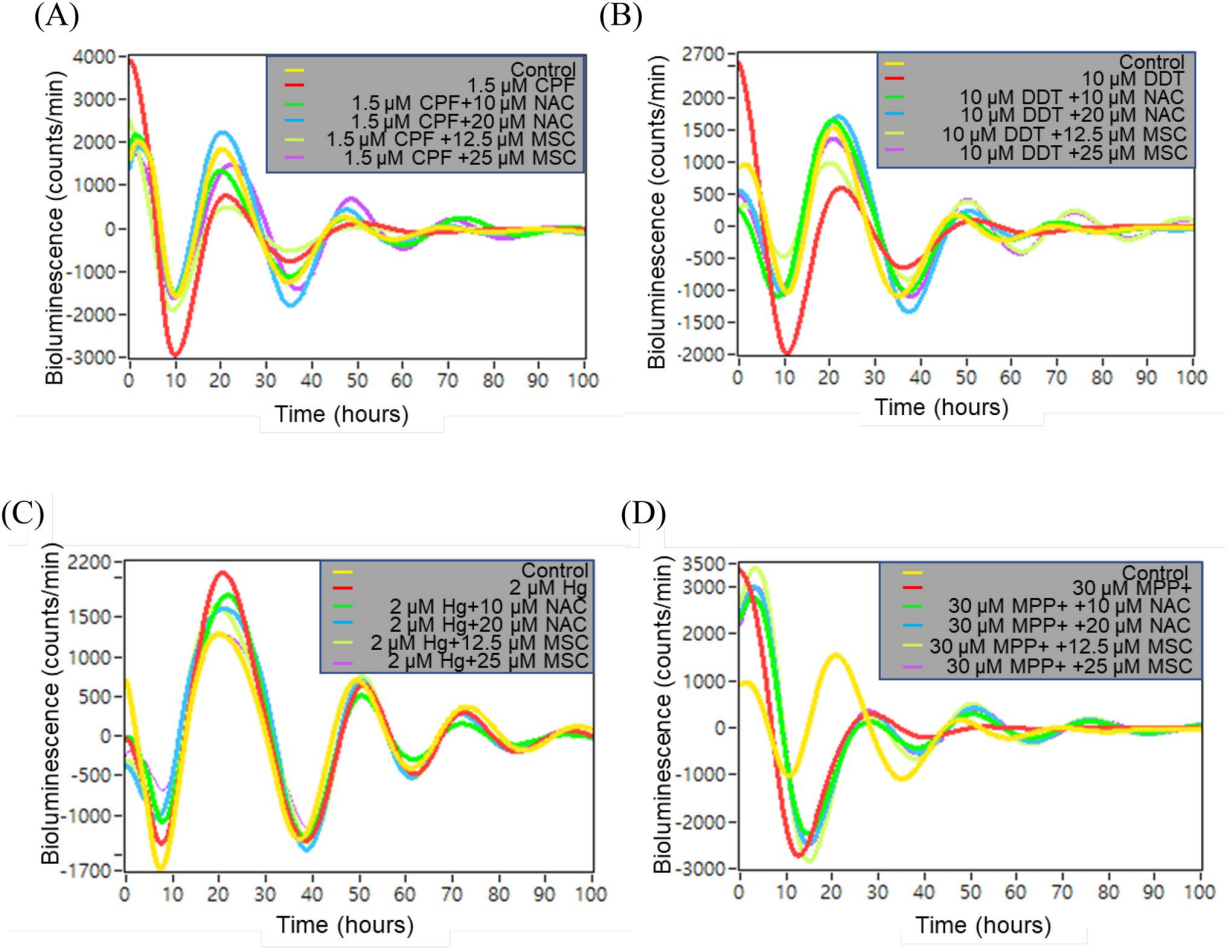
Antioxidants restore cellular circadian rhythms disrupted by chemicals in U87 MG/*PER*2-dLuc cells in vitro. **A** MSC and NAC restore the cellular circadian rhythm disrupted by CPF in U87 MG/*PER*2-dLuc cells. In cells treated with **B** DDT, **C** MHg, or **D** MPP + , MSC and NAC restore the cellular circadian rhythm to a certain extent. *CPF* chlorpyrifos, *CPX* ciprofloxacin, *DDT*,4,4′-dichlorodiphenyltrichloroethane, *MHg* methylmercury, *MPP +* 1 -methyl-4-phenylpyridinium, *MSC* methylselenocysteine, *NAC* *N*-acetyl-l-cysteine, *PMX* polymyxin B

Although treatment with 1.5 µM CPF abolished the cellular circadian rhythm, as indicated by the disappearance of the second luminescence peak in the reporter activity assay, both 20 µM NAC and 12.5 µM (or 25 µM) MSC restored the circadian rhythm in CPF-treated cells and helped maintain a normal cellular rhythm (Fig. 4A). Similarly, co-treatment with NAC or MSC restored the cellular rhythm disrupted by 10 µM DDT to a normal rhythm (Fig. 4B). Co-treatment with NAC and MSC reduced the first high-altitude peak induced by 2.0 µM MHg (Fig. 4C). These antioxidant agents could restore the MPP + -disrupted rhythmicity; however, the restored rhythm was not similar to that of control cells (Fig. 4D). Conversely, co-treatment with PMX and CPF and antioxidants failed to consistently restore disrupted rhythms (data not shown).

### Changes in nuclear size induced after chemical treatment for 24 and 72 h

**Fig. 5:**
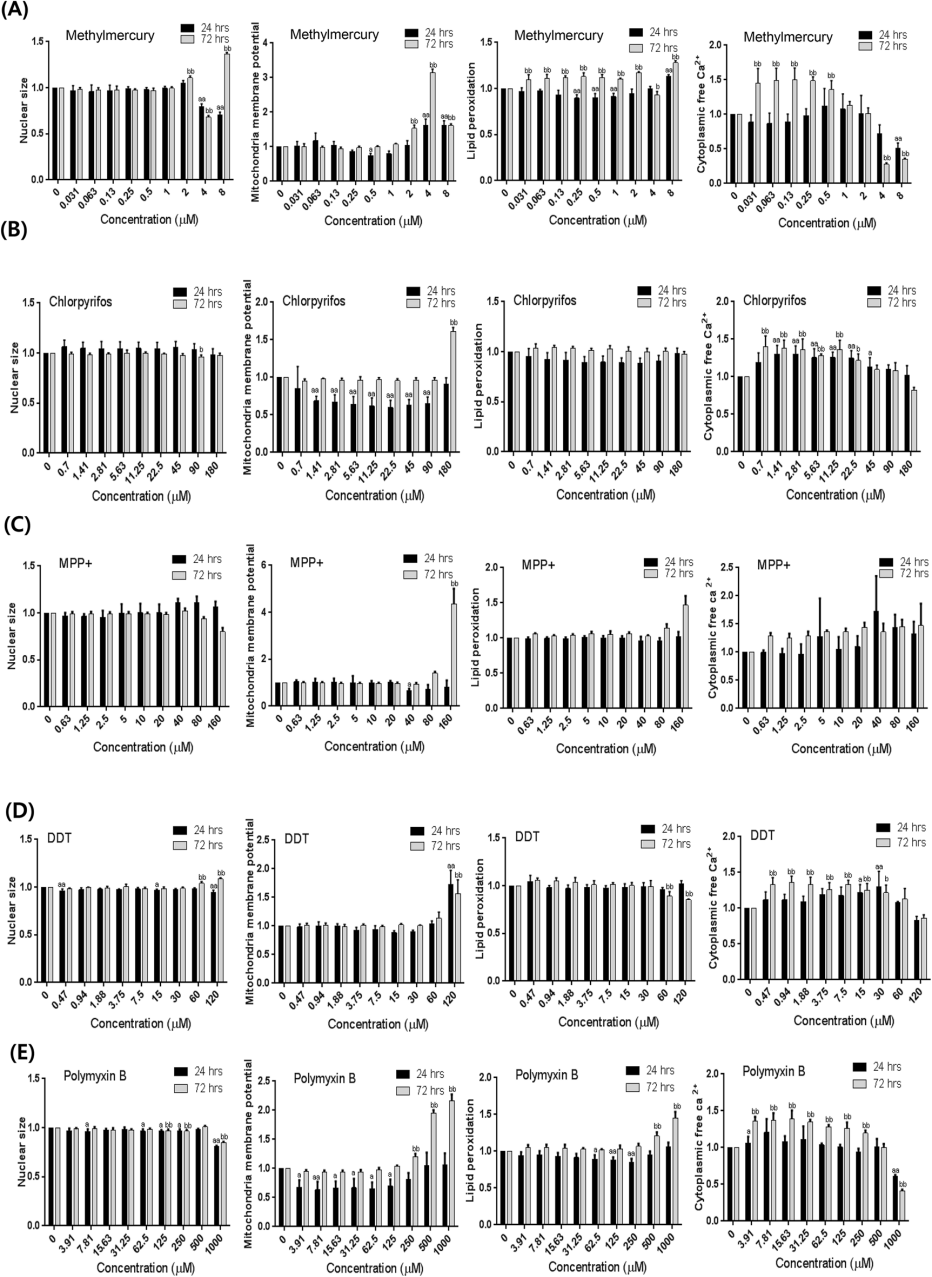

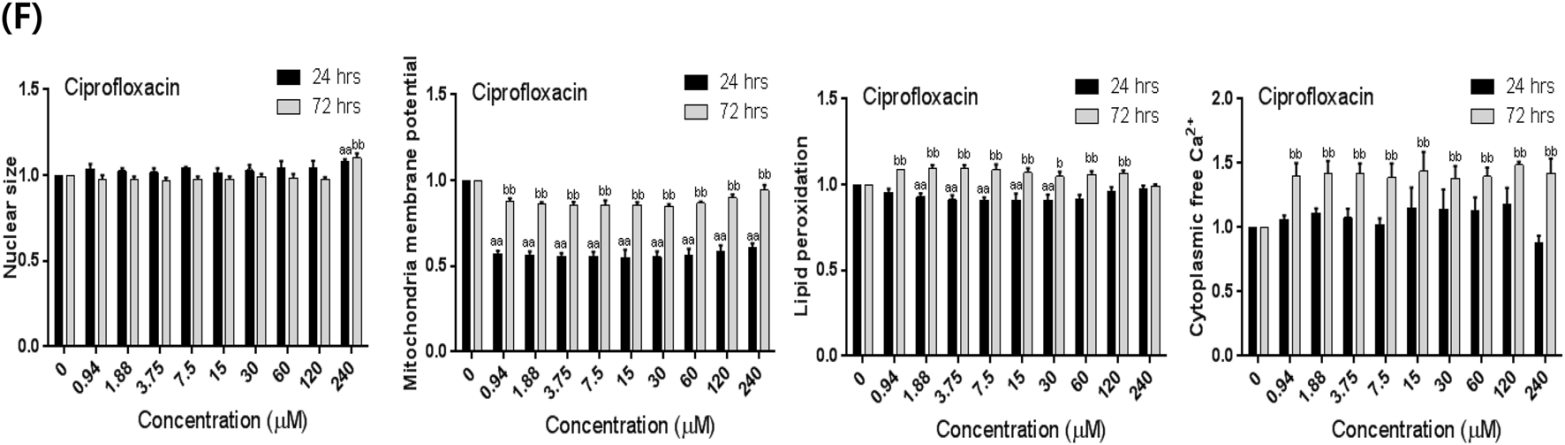
Relative changes in cytotoxic parameters in HCS in U87 MG/*PER*2-dLuc cells treated with the neurotoxicants for 24 and 72 h. U87 MG/*PER*2-dLuc cells were treated with **A** MHg, **B** CPF, **C** MPP + , **D** DDT, **E** PMX, or **F** CPX for 24 or 72 h, and then subjected to HCS assay as described in Materials and Methods. In the legend, the indicators (Y-axis) measured for each administered substance (**A**–**E**) are explained from left to right: nuclear size, MMP, lipid peroxidation, and Ca^2+^ levels as the ratio of the test substance to the vehicle control. Values are the mean ± standard deviation (SD) of triplicates. “a” and “aa” indicate *p* < 0.05 and *p* < 0.01, respectively, vs. 0 µM for 24 h. “b” and “bb” indicate *p* < 0.05 and *p* < 0.01, respectively, vs. 0 µM for 72 h. CPF, chlorpyrifos; CPX, ciprofloxacin; DDT, 4,4′-dichlorodiphenyltrichloroethane; HCS, high-content-screening; MHg, methylmercury; MMP, mitochondrial membrane potential; MPP + ,1 -methyl-4-phenylpyridinium; PMX, polymyxin B

To examine the effects of the chemicals on the nuclear size of U87 MG/*PER*2-dLuc cells, Hoechst 33342 was used as a molecular probe to detect the area of nuclear fluorescence in treated cells when compared with that in non-treated control cells. Relative changes in the nuclear size indicated adverse effects associated with cytotoxicity. MHg had dual effects, with a slight increase followed by a significant decrease in nuclear size at 4–8 µM of MHg (above the IC_50_) after 24 h of treatment (*p* < 0.01). Moreover, MHg exerted substantial hormesis, with moderate biphasic changes in nuclear size observed at 2–8 µM of MHg (around the IC_50_) after 72 h of treatment (Fig. 5A). After 72 h of treatment, MPP + could sequentially reduce nuclear size at concentrations of 80–120 µM (around the IC_50_) (Fig. 5C). After 24 h of treatment, DDT significantly reduced the nuclear size at concentrations at 120 µM (above the IC_50_) (*p* < 0.01) (Fig. 5D).

### Neurotoxicant treatment for 24 h reduced MMP levels

To examine the effects of selected chemicals on the MMP of cells, TMRM was used as a molecular probe to detect the MMP, and the fluorescence intensity of treated cells was compared with that of non-treated control cells. A relative reduction in TMRM fluorescence greater than that of negative controls indicates a decrease in the MMP, a well-known adverse effect associated with cytotoxicity. Following 24 h of treatment, we observed that all examined chemicals could significantly reduce MMP at concentrations substantially lower than the IC_50_ value (*p* < 0.01 or *p* < 0.05) (Fig. 5A–F). Cytotoxicity, as indicated by a decrease in the MMP, was observed at the lowest concentrations, in ascending order: 0.5 µM for MHg, 0.94 µM for CPX, 1.41 µM for CPF, 3.91 µM for PMX, 15 µM for DDT, and 40 µM for MPP +  (Fig. 5). Conversely, MMP levels were not significantly altered by most chemicals following 72 h of treatment; one exception was CPX, which could significantly decrease MMP levels at concentrations ≥ 0.94 µM even after 72-h treatment (*p* < 0.01). Interestingly, we observed that certain capable of reducing MMP levels showed a tendency for biphasic reactions, decreasing and then increasing MMP with increasing concentrations; however, this observation was considered to be hormesis (O'Brien et al. 2006).

### Treatment with neurotoxicants for 24 and 72 h reduced calcium ion levels

Next, the Fluo-4 AM probe was used to evaluate the effects of selected chemicals on the endoplasmic reticulum. An increase in green fluorescence indicates an increase in the intracellular free Ca^2+^ concentration. At concentrations ranging from 0.7 to 45 µM, CPF increased the intracellular Ca^2+^ level at both the 24- and 72-h timepoints (Fig. 5B). Treatment with MPP + at concentrations of 40 and 0.63 µM increased intracellular Ca^2+^ levels at 24- and 72-h timepoints, respectively (Fig. 5C). However, treatment with the other chemicals did not significantly enhance intracellular Ca^2+^ levels after 24 h of treatment (Fig. 5A, E and F). Following treatment for 72 h, the other chemicals (i.e., other than MPP + and CPF) could enhance intracellular Ca^2+^ levels at concentrations lower than the IC_15_ value (Fig. 5A, D–F). At 72 h, intracellular Ca^2+^ levels were elevated at concentrations above 0.03 µM for MHg, 0.47 µM for DDT, and 0.94 µM for CPX (Fig. 5A, D and E).

### Treatment with chemicals for increased lipid peroxidation primarily after 72 h

BODIPY 665/676 was used as a molecular probe to examine the oxidative degradation of cell membranes. Increased red fluorescence indicates enhanced lipid peroxidation induced by oxidative stress (Choi et al. 2007). As shown in Fig. 5, cytotoxicity, as indicated by an increase in lipid peroxidation, was observed in ascending order at serial low concentrations. Treatment with most chemicals for 24-h did not significantly alter the lipid peroxidation levels. However, MHg significantly reduced lipid peroxidation levels at concentrations above the IC_50_ value, 8.0 µM, 24 h after treatment.

Treatment with certain chemicals could significantly increase lipid peroxidation levels 72 h after treatment. Specifically, chemically treated cells showed increased lipid peroxidation at concentrations above 0.031 μM for MHg, 160 µM for MPP + , and 0.94 µM for CPX after 72 h of treatment (Fig. 5).

### Association between disruption of cellular circadian rhythm and toxic indicators of cellular function

Table 1 summarizes the effects of all examined compounds on cellular circadian rhythm and individual toxic indicators of cellular function. The LOAELs of all compounds for the cellular circadian rhythm were below or approximated the IC_15_ value, except that of CPX (40 µM) for circadian disruption, which was in the range of LC_15_–LC_50_ (20–98 µM). These results revealed that the nuclear size was relatively stable, especially at low doses of each chemical. However, nuclear size was significantly altered (increased or decreased) by most chemicals at high doses, especially MHg, DDT, and PMX, after both 24 or 72 h of treatment (*p* < 0.01 or *p* < 0.05). For examined chemicals, the LOAEL for circadian rhythm disruption was slightly higher than that of most cytotoxicity indices detected using HCS. The LOAEL for MMP reduction in the first 24 h was slightly lower than that for circadian disruption and the closest to it. For CPF and DDT, the LOAEL for calcium ions closely approximated the LOAEL for circadian disruption at 24 h. Considering MHg, MPP + , and CPX, the LOAELs for lipid peroxidation were increased at 72 h.

## Discussion

Herein, we evaluated the applicability of an in vitro circadian reporter assay using human glioblastoma cells (U87 MG/h*PER*2-dLuc) to assess the potential toxicity of environmental chemicals. The combined application of the multiparametric HCS method and the in vitro circadian reporter system can provide a mechanistic basis for effortless neurotoxicity screening based on different cellular functions. Additionally, we evaluated whether a cellular bioluminescence assay could be employed as an early and sensitive biomarker for evaluating the toxicity of neurotoxicants. Comparing concentrations that induced changes in cellular circadian rhythm with those that altered different mechanistic endpoints, we gained insight into the association between cellular rhythm dysfunction and cytotoxicity in neural cells. We found that chemicals well-known to induce neurotoxicity, including a heavy metal (MHg), pesticides (CPF, MPP + , and DDT), and antibiotic drugs (CPX and PMX), disrupted circadian rhythm by altering the amplitude and/or advancing the phase with continued high amplitude. Moreover, our findings revealed that a multiparametric cellular image-based HCS method can be utilized to assess the cytotoxic effects of environmental toxicants based on indicators of cellular functions in U87 MG/h*PER*2-dLuc. Assessing cytotoxic endpoints between the cellular circadian rhythm and indicators of cellular functions suggests that the circadian rhythm disruption assay correlates with the mechanism of cytotoxicity. Therefore, the assay sensitivity to potential cytotoxic effects can be evaluated and compared.

We demonstrated that chemicals could disrupt the circadian rhythm of *PER*2 expression and parameters of HCS, such as NC, MMP, CA, and LP. This finding suggests that the in vitro circadian reporter cell system is associated with the molecular endpoint of cytotoxicity and possesses the characteristics of conventional in vitro cell-based toxicity assays. Thus, the circadian rhythm of *PER*2 expression may be a useful biomarker. Heavy metals and pesticides are more cytotoxic than antibiotics, even at lower concentrations, severely disrupting normal, synchronized circadian rhythms. When the minimum concentration for a disturbed circadian rhythm of *PER*2 was compared with that in the HCS assay at 24 or 72 h, the minimal disruptive concentration of tested chemicals was similar to the concentration that significantly altered MMP at 24 h, indicating that disruption of the circadian expression of *PER*2 is related to the molecular indicator of cytotoxicity. Notably, we observed that co-treatment with antioxidant chemicals could prevent or restore cellular circadian rhythm disruption induced by MHg, CPF, MPP + , and DDT, indicating that oxidative stress-mediated MMP reduction could be a highly relevant factor associated with circadian rhythm disruption. However, circadian rhythm disruption mediated by PMX and CPX was not closely or consistently associated with cytotoxicity endpoints. Moreover, co-treatment with PMX or CPX and antioxidants afforded inconsistent or unreproducible results. Accordingly, further studies are warranted to confirm the effects of antioxidants on the actions of PMX and CPX. In addition, in vitro assessments of U87 MG/*PER*2-dLuc cells in the presence of neurotoxins and antioxidants could be valuable in identifying the defense mechanism of antioxidants.

We tested the values of cytotoxicity parameters for HCS. The chronological, biological neurotoxicity following different durations of chemical exposure revealed a distinct trend at 24 and 72 h. A reduction in the MMP level during the early phase (24 h) of the circadian period is assumed to be the earliest and most sensitive indicator of cellular circadian rhythm disruption, whereas elevated intracellular Ca^2+^ levels and lipid peroxidation appear to be secondary markers of the early/late or the late phase (24/72 h or 72 h), respectively. These results are similar to the liver cell model, which initially showed a reduction in NC and MMP, followed by a gradual enhancement in Ca ions, i.e., a time course of cytotoxic change. This study shows that the cytotoxic effect increases over time above a threshold drug concentration (O'Brien et al. 2006).

**Fig. 6:**
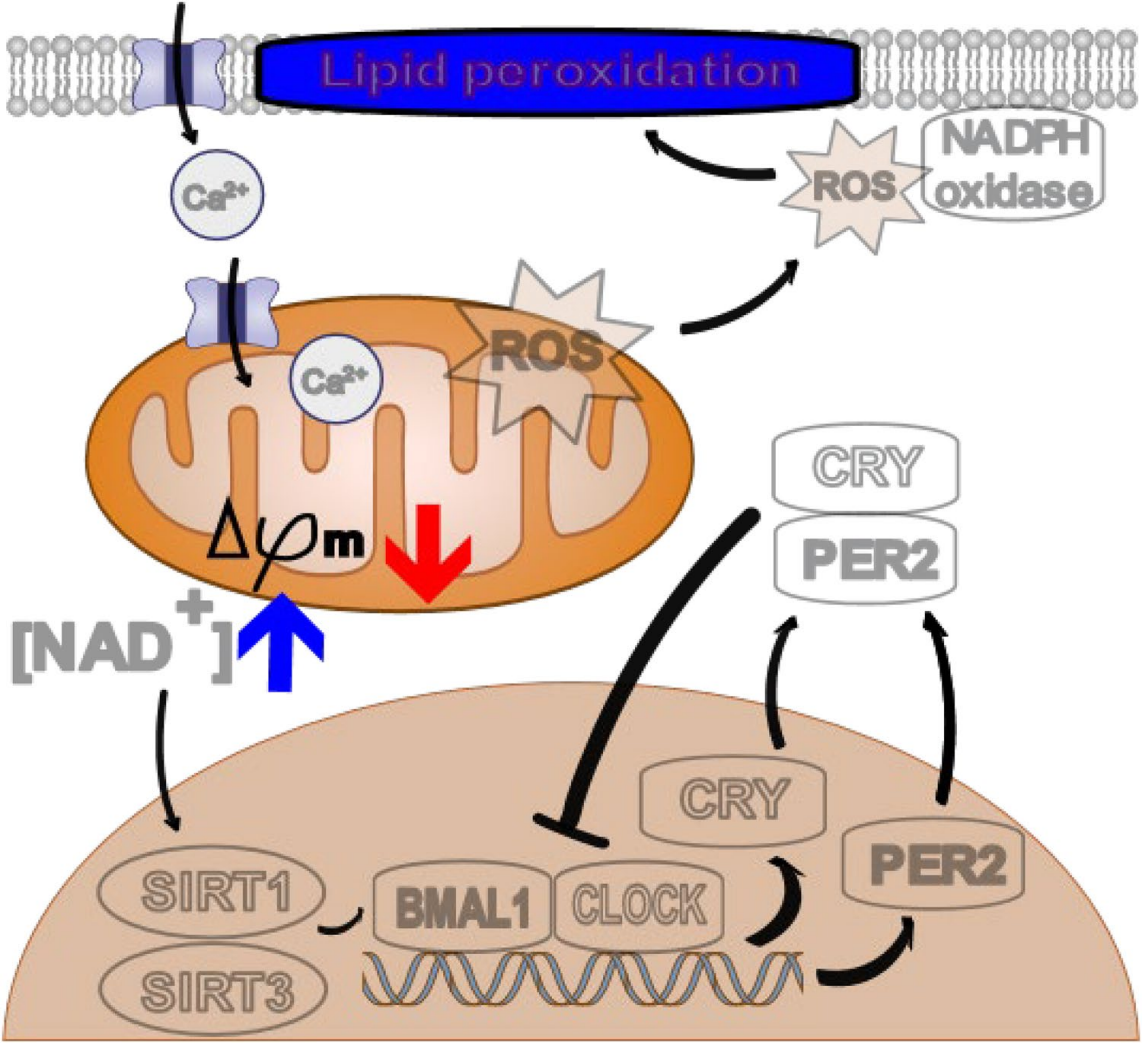
Schematic diagram illustrating the putative mechanistic relationship between circadian genes and cytotoxicity markers in mitochondria. The decrease in MMP and increase in calcium ions levels via the mitochondria-circadian rhythm axis result in circadian rhythm disruption and ROS generation via cell damage, thereby stimulating lipid peroxidation. ROS, reactive oxygen species

Furthermore, DDT- or CPF-mediated increased intracellular calcium concentrations [Ca2 +]_i_ via calcium channel inhibition (Meijer et al. 2014; Stevens et al. 2011) may indirectly disrupt the circadian rhythm by increasing oxidative stress, as shown in the mechanistic diagram. In addition, elevated lipid peroxidation, which is induced by oxidative stress and calcium ions, is likely associated with mitochondrial damage, disrupting the circadian rhythm (Fig. 6). Collectively, the findings of the current study suggest that circadian rhythm disruption is associated with cytotoxicity, which progresses from abnormal MMP to oxidative stress and Ca^2+^ influx (Baev et al. 2022). It is well-established that abnormalities in mitochondrial function induce excessive ROS generation (Guo et al. 2013). Therefore, further mechanistic studies are warranted. The results of in vitro bioluminescence assays using circadian reporter cells may exhibit similar sensitivity to predictors of HCS cytotoxicity assays, potentially indicating a close relationship between cellular rhythm disturbances and cytotoxic mechanisms.

The putative interdependent function of *PER*2 and mitochondria suggests that molecular pathways in mammalian circadian clocks respond to chemicals, leading to dysfunctions, such as reduced MMP and increased calcium ion influx, during the early cell damage process and subsequent oxidative stress. Typically, neural mitochondrial dysfunction and ROS affect the accumulation of lipid droplets in glial cells (Liu et al. 2015). Furthermore, the SIRT3-NAD^+^ axis reportedly serves as a metabolic link between mitochondrial function and the circadian rhythm (de Goede et al. 2018; Manella and Asher 2016; Sardon Puig et al. 2018). Specifically, the interdependence of circadian rhythm disruption and reduced MMP induced by neurotoxic agents was confirmed by an increase in MMP owing to the restoration of the disrupted circadian rhythm (Kenche et al. 2021). In the current study, changes in *PER*2 in all tests showed a clear change in the circadian rhythm during the initial phase of the assay, along with a decrease in the MMP. Furthermore, we found that antioxidants (MSCs and NACs) known to reduce neurotoxicity could prevent the loss of cellular circadian rhythms or even restore normal patterns. These results suggest that antioxidant-mediated restoration of the disrupted circadian rhythm is associated with the normalization of the MMP and preventive effect on oxidative stress.

In addition, we identified other associations between alterations in circadian rhythm and intracellular Ca^2+^ following chemical treatment. The circadian rhythm phase was prolonged when CPF and DDT increased intracellular Ca^2+^ levels at 24 and 72 h, while CPX increased Ca^2+^ only at 72 h. These results revealed that neurotoxic chemicals could increase the movement of Ca^2+^ across the neuronal plasma membrane, enhancing intracellular calcium concentrations [Ca2 +]_i_ (Bondy 1989). Considering persistent molecular rhythms in the suprachiasmatic nucleus, periodic transmembrane flux of Ca^2+^ due to circadian changes in membrane potential is an important process that functions as a circadian pacemaker (Lundkvist et al. 2005). Therefore, follow-up investigations are warranted to examine the relationship between the cellular circadian rhythm and elevated levels of cytosolic calcium.

In summary, our results suggest that the in vitro circadian reporter bioluminescence cell assay is highly sensitive and could allow the assessment and evaluation of neurotoxicity at trace levels of environmental chemicals, pesticides, and drugs. Furthermore, in this model system, disrupting the circadian rhythm of *PER*2 using a test item capable of inducing environmental toxicity was associated with mitochondrial toxicity, calcium ions, and oxidative stress in human neural cells (Meyer et al. 2018).

Moreover, we demonstrated that antioxidants could recover or prevent circadian rhythm disruption. Although the relationship between oxidative stress and cellular rhythm has been explored (Mezhnina et al. 2022; Sun et al. 2020), molecular mechanisms underlying the restorative and preventive effects of antioxidants on disrupted cellular rhythms need to be elucidated. If specific molecular mechanisms were identified and confirmed to be active in existing cytotoxicity evaluation methods, a high-throughput circadian rhythm neurotoxicity assay for neurotoxicity screening would be an efficient and appropriate alternative assay for in vivo toxicity tests.

## Data Availability

The datasets used and/or analysed during the current study available from the corresponding author on reasonable request.
